# A multidisciplinary approach to study the reproductive biology of wild prawns

**DOI:** 10.1038/s41598-017-16894-1

**Published:** 2017-12-01

**Authors:** L. Bolognini, F. Donato, A. Lucchetti, I. Olivotto, C. Truzzi, B. Randazzo, M. Antonucci, S. Illuminati, F. Grati

**Affiliations:** 1 National Research Council – Institute of Marine Science, Largo Fiera della Pesca, 60125 Ancona, Italy; 2 Department of Life and Environmental Sciences, Università Politecnica delle Marche, Via Brecce Bianche, 60131 Ancona, Italy

## Abstract

This work aims to provide deeper knowledge on reproductive biology of *P. kerathurus* in a multidisciplinary way. Upon 789 examined females, 285 were found inseminated. The logistic equation enabled to estimate the size at first maturity at 30.7 mm CL for female. The Gono-Somatic Index (GSI) showed a pronounced seasonality, ranged from 0.80 ± 0.34 to 11.24 ± 5.72. Histological analysis highlighted five stages of ovarian development. Gonadal fatty acids analysis performed with gas chromatograph evidenced a pronounced seasonal variation; total lipids varied from 1.7% dry weight (dw) in Winter, to 7.2% dw in Summer. For the first time, a chemometric approach (Principal Component Analysis) was applied to relate GSI with total lipid content and fatty acid composition of gonads. The first two components (PC1 and PC2) showed that seasonality explained about 84% of the variability of all data set. In particular, in the period February-May, lipids were characterized by high PUFAs content, that were probably utilized during embryogenesis as energy source and as constituent of the cell membranes. During the summer season, gonads accumulated saturated FAs, that will be used during embryogenesis and early larval stages, while in the cold season total lipids decreased drastically and the gonad reached a quiescent state.

## Introduction

The Mediterranean Sea is one of the most important marine regions in the world due to its high biodiversity that supports a locally significant and diversified fishing^1^. In this basin fishing activities play an important role from a social as well as an economic point of view. However, recent stock assessments have certified the general decline of resources in this area, where most of the stocks are classified as exploited above Fishing Mortality Sustainable Yield (FMSY^2^) or overfished^3,4^ because of an unsustainable fishing pressures.

In the last ten years, in countertendency with a general decline, the Decapod Crustacean species landings significantly increased, and a key role is played by the caramote shrimp (*Penaeus kerathurus*), that is a high priced shrimp, due to its large size and the quality of its meat^5^.

*P. kerathurus* is a large Mediterranean autochthonous shrimp that lives on soft bottoms of the continental shelf, usually at less than 60 meters depth; juveniles enter lagoons and are common on coastal grounds in late summer and autumn^6–8^. It is mainly caught by bottom trawling and, to a less extent, by gillnetters^9^. Nowadays, it is a highly valuable fishery resource in the Northern and central Adriatic Sea (GSA-17), with annual landings estimated around 500 tons, and a peak in the last quarter of the year, when the new generation of shrimps, born in summer, move offshore and is fully recruited to the fishery^8^.

In the same period, a marked reduction of the landings of another Decapod Crustacean species, the Norway lobster (*Nephrops norvegicus*), was recorded^8^.

One of the key factors affecting the opposite trend in the abundance of different species is the reproductive biology. An understanding of the reproductive biology of a species is a central aspect to provide sound scientific advice for fisheries management. The success of reproductive biology determines productivity and therefore a population’s resilience to fisheries exploitation or other human perturbations. The importance of quantifying productivity in terms of reproductive potential (RP) and recruitment, as well as the difficulty in doing so, have long been recognized^10,11^. The understanding of the dynamics of a stock is crucial to know the relation between size and reproduction event, such as mating and sexual maturity^12^.

One of the most important factors determining the RP of a species is the reproductive pattern, which is essential for future domestication of the species in local waters^13^.

Life history parameters of *P. kerathurus*, such as maturity at size or age, sex ratio, fecundity and spawning time and duration, vary between populations, but they can also vary temporally within a population, altering the RP over the time^14^. In this regards, standardization criteria for evaluating sexual maturity and classify maturity stages in Crustaceans were strongly recommended by the International Council for the Exploitation of the Sea^15^ and General Fisheries Commission for the Mediterranean (GFCM). ICES Expert Group (2010)^15^ also highlighted the importance of histology as a tool for obtaining the highest accuracy in these type of studies^12^.

The reproductive cycle of penaeids is affected by many factors as seasonal rainfall, temperature regime and depth, exhibiting a rather complex life history^16,17^. Typically, tropical and subtropical penaeids exhibit a bimodal seasonal spawning pattern, while spawning becomes unimodal in penaeids that inhabit temperate zones, characterized by only one well-defined recruitment period^16,18,19^.

During the reproductive cycle of decapod crustaceans, the ovarian development is accompanied by changes in color and size^20–22^. In some species, these changes in color are easily visualized and result from differences in carotenoid content, which play an important role during embryogenesis^23–25^.

In recent years, increased attention has been reported to the ovarian development of penaeid shrimp, and the macroscopic and histological characterization have been studied for various species, such as *Melicertus japonicus* ^26^, *Melicertus vannamei* ^27^, *Melicertus semisulcatus* ^28^, *Melicertus kerathururs* ^29^, *Melicertus monodon* ^30,31^, *Litopenaeus setiferus* ^32^, *Penaeus indicus* ^33^, *Melicertus aztecus* ^34^, *Fenneropenaeus merguiensis* ^35^ and *Melicertus plebejus* ^36^.

Many studies highlighted the crucial role of lipid deposition in the ovary during natural and induced sexual maturation of various shrimp species^37–44^. Shrimp juveniles may have a limited capacity for *de novo* phospholipid synthesis^45^ and an inability to synthesize cholesterol^46^.

Information on the quantitative changes that occur in the different lipid classes and their fatty acids during the maturation period are scarce. Teshima and Kanazawa^38^ indicated that large quantities of lipids were necessary for the development of *P. japonicus* ovaries, and the amounts of ovarian lipids increased remarkably with increasing gonado-somatic index (GSI) values. These authors highlighted a quantitative ovarian lipid increasing during the sexual maturation, conversely their qualities did not vary so remarkably in the same prawn species.

The present study focuses on the gametogenic cycle of caramote prawn in the Northern and central Adriatic Sea. The current study aims, for the first time in Mediterranean Sea, to characterize the reproductive event in *P. kerathurus* using a multidisciplinary approach including biometrics (carapace length, body weight, gender and gonado-somatic index); morphological (ovarian histology) and biochemical approaches such as lipid composition profile of the ovary during the annual spawning season.

## Results

**Figure 1:**
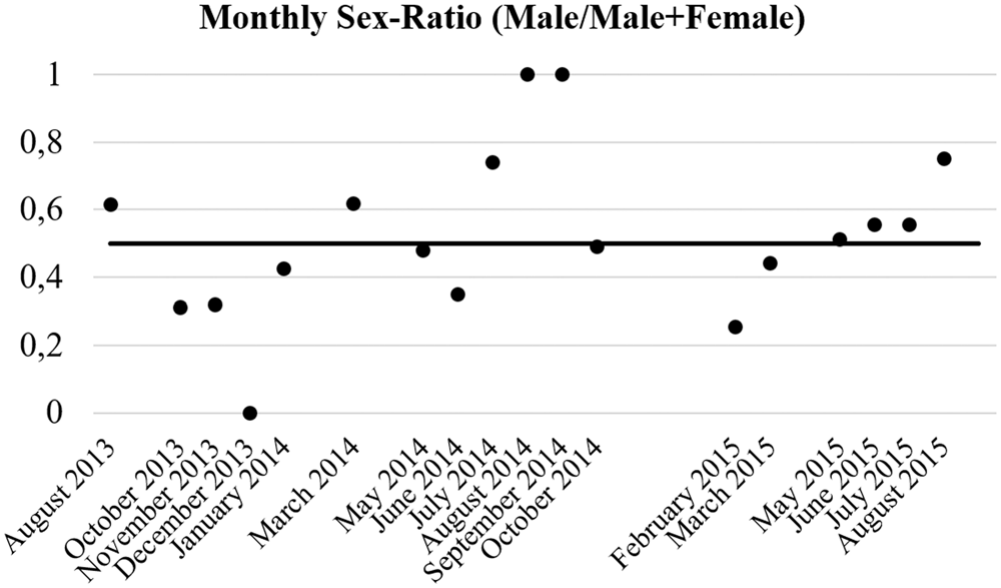
Total and monthly sex-ratio of P. kerathurus (males/males+females) population, from August 2013 to September 2015.

From August 2013 to September 2015 a total of 1482 *P. kerathurus* specimens were collected; 790 of these were females (53%) and 692 were males (47%). The monthly analysis of sex-ratio showed an average value of 0.52 (±0.24) males/males + females; in general, the sex ratio was biased towards female in the population (sex ratio = 0.466; Chi-square test of goodness of fit: χ^2^ = 6.480, df = 1, p = 0.0109; Fig. 1).

**Figure 2:**
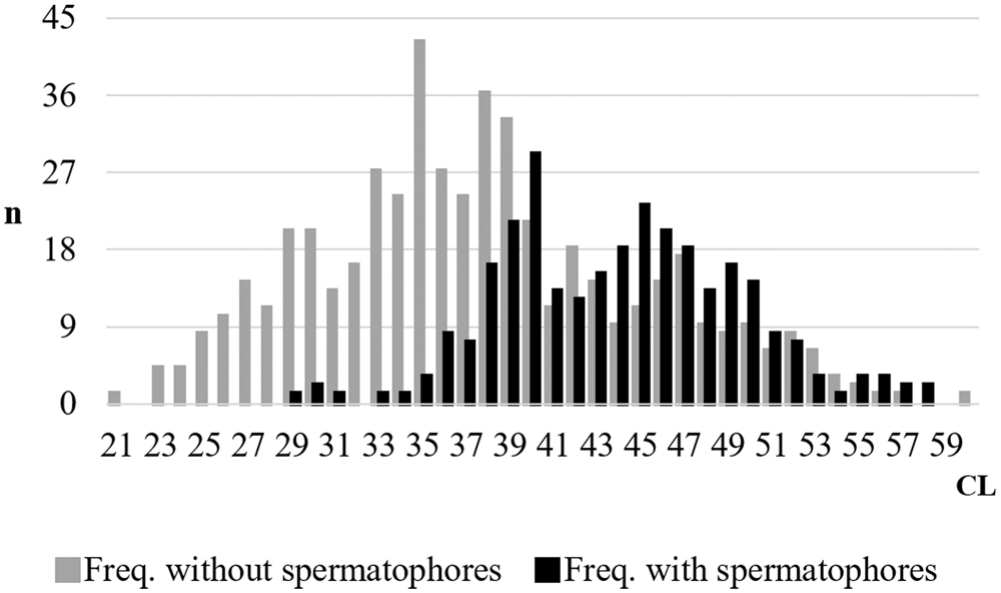
Length-frequency distribution of *P. kerathurus* females with and without spermatophores.

Upon 789 examined females, 285 were found to be inseminated (36.12%). Their size ranged between 29 and 58 mm CL with a mean (±sd) of 44.31 ± 5.28 mm. The not inseminated females showed a wider size range, with dominant smaller size classes with a mean of 37.90 ± 7.28 mm CL, and ranged between 21 and 60 mm CL (Fig. 2). The CL medians and the size frequency distribution of the two groups (inseminated and not inseminated females) showed significant differences (Mann-Whitney test for equal medians: N = 789, p = 1.5966 × 10^−35^; two-sample Kolmogorov-Smirnov test for equal distributions: N = 789, p = 1.5571 × 10^−34^).

**Figure 3:**
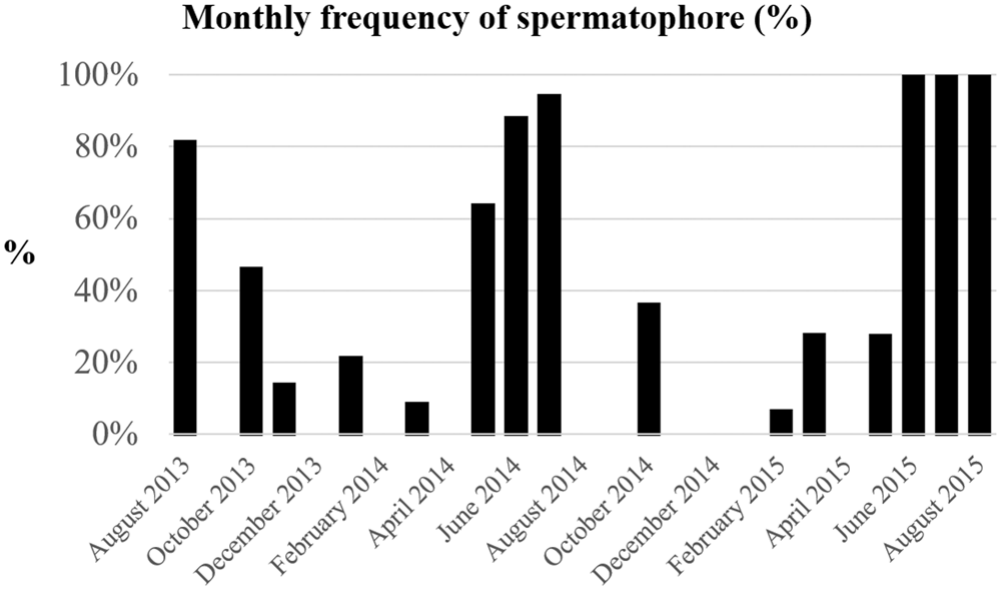
Monthly frequency of spermatophores (%) in P. kerathurus females, from August 2013 to September 2015.

Inseminated females were more frequent during summer season, in particular June, July and August. In June 2014 inseminated females were collected in 87% of samplings, while they were always sampled in June and July 2015. The presence of inseminated females decreased in October (44.8% in 2013 and 34.8% in 2014) and in May (62.5% in 2014 and 26.1% in 2015). Values under 26% of inseminated females were recorded in the remaining months (Fig. 3). The analysis of variance (one-way ANOVA) tested for the monthly sex ratio and monthly frequency of spermatophore did not highlight inter-annual variation during the sampling season (from August 2013 to September 2015; p-value respectively p = 0.09287 and p = 0.6222).

In August the trawling ban, in place since 1987, did not allow to collect a reasonable number of caramote prawns.

**Figure 4:**
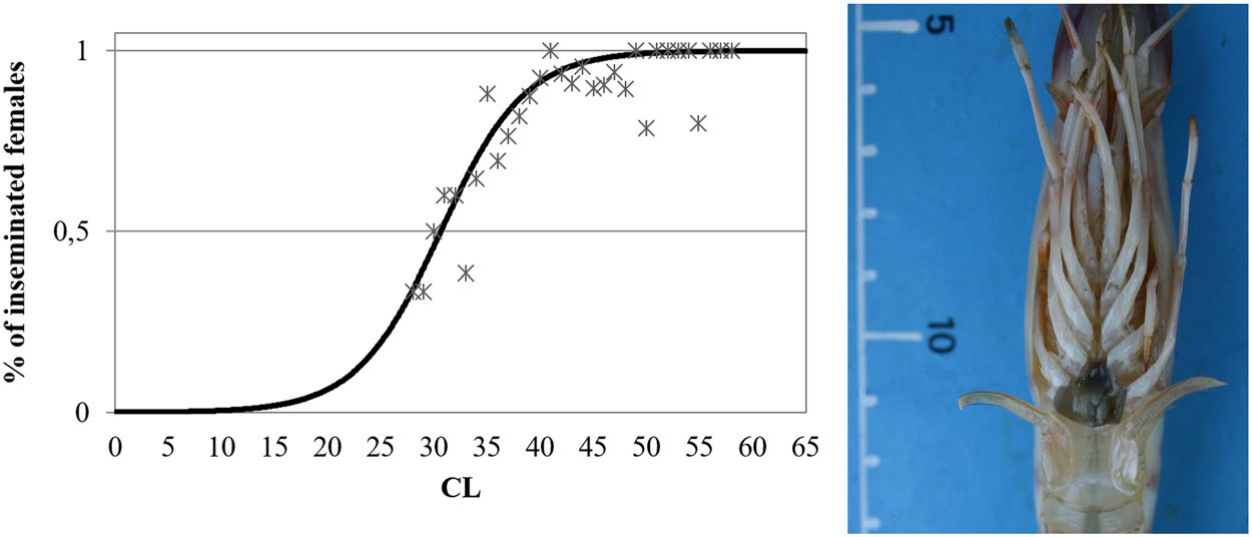
Logistic curve for the estimation of length at first maturity (CL, mm; on the left) based on the occurrence of insemination in P. kerathurus females (on the right). Logistic equation: y = 0.59515/(1 + 5.3185E09*exp(−0.5961x).

The logistic equation (King, 1995) enabled to estimate the size at first maturity at 30.7 mm CL for female (Fig. 4).

**Figure 5:**
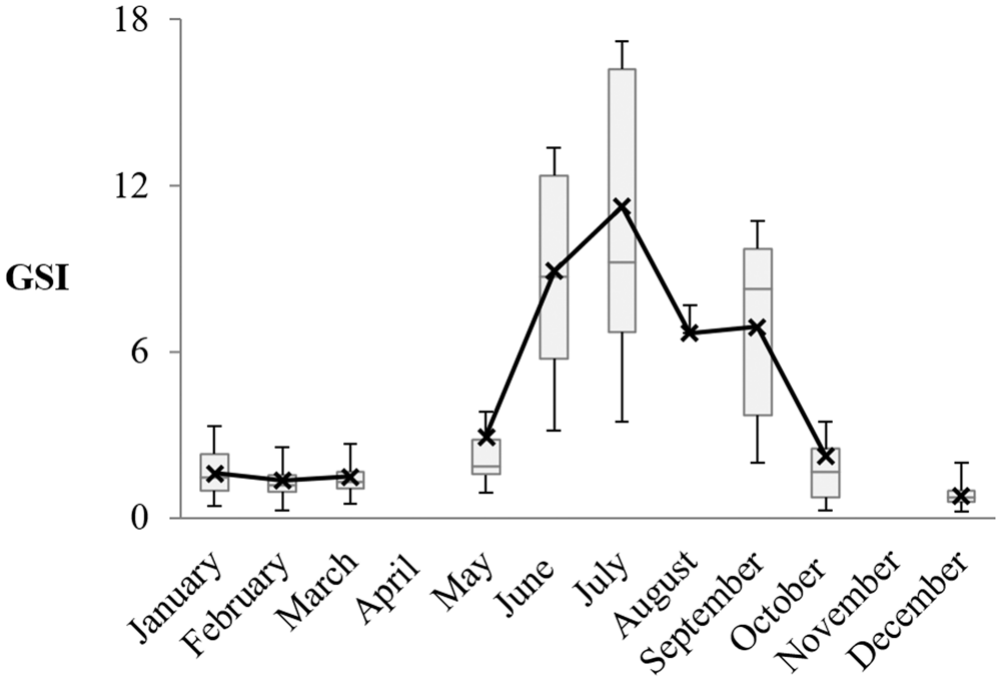
Box and Whiskers plot of monthly variation of GSI of P. kerathurus females.

The Gonado-Somatic Index obtained from monthly-pooled data, showed a pronounced seasonality. The minimum value was recorded on December (0.80 ± 0.34) and in general, under 1.6 in winter months; a very pronounced increased values were observed since May (2.93 ± 2.45) with a pick in July (11.24 ± 5.72), followed by a decrease until October (2.23 ± 2.70; Fig. 5).

### Histology

According to literature^29^, five stages of ovarian development of *P. kerathurus* were identified, based on oocyte stage.

#### Stage I (Previtellogenic)

**Figure 6:**
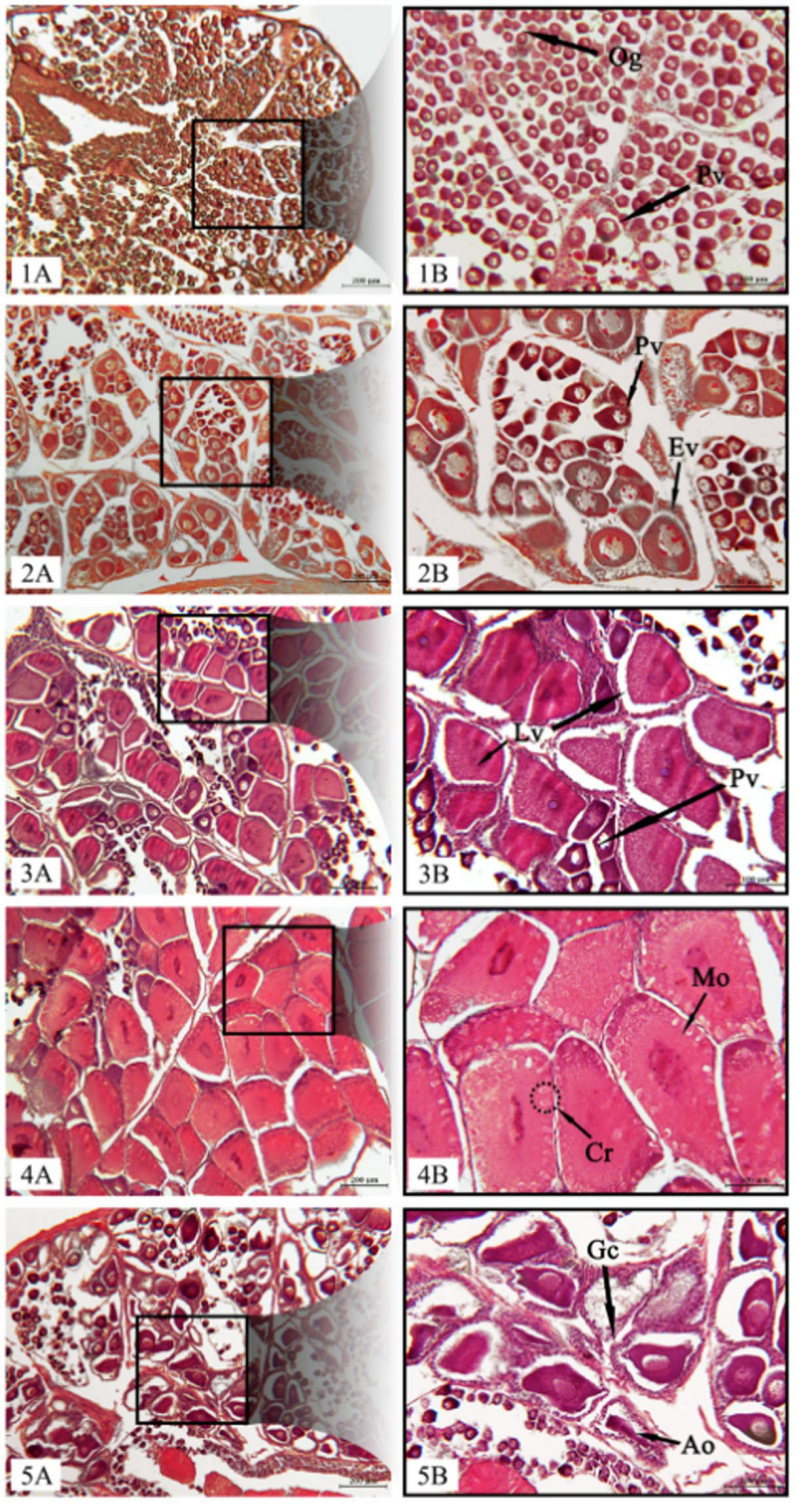
Transverse histological sections of female gonads of P. kerathurus (scale bars: 200 μm on the left: **A**, 100 μm on the right: **B**). (1A–1B) stage 1: ovary showing only oogonia (Oo) and previtellogenic (Pv) oocytes; (2A–2B) stage 2: ovary with previtellogenic and early vitellogenic (Ev) oocytes; (3A–3B) stage 3: ovary with previtellogenic and latevitellogenic (Lv) oocytes; (4A–4B) stage 4: ovary showing previtellogenic and mature oocytes (Mo) and cortical rods (Cr); (5A–5B) stage 5: ovary showing atretic oocytes (Ao) and granulosa cells(Gc).

Macroscopically gonad are very small and not visible through the exoskeleton. The ovary is white translucent, contained oogonia (Oo) and previtellogenic oocytes (Pv). The size range of previtellogenic oocytes is from 34 to 53 μm in diameter (Supplementary Fig. S3, Fig. 6–1A/B).

#### Stage II (Early Vitellogenic)

Developing ovaries are flaccid and white-ivory coloured. They consist of previtellogenic and larger early vitellogenic oocytes (Ev, 80–100 μm). In vitellogenic oocytes, the cytoplasm is more basophilic and the nucleus is surrounded by a few nucleoli (Supplementary Fig. S3, Fig. 6–2A/2B).

#### Stage III (Late Vitellogenic)

Fresh ovaries are large and visible through the exoskeleton and yellow to pale-olive. Ovarian follicols contain previtellogenic oocytes and late vitellogenic oocytes (Lv, 90–150 μm) located at the periphery, filled with small scattered yolk vesicoles (Supplementary Fig. S3, Fig. 6–3A/3B).

#### Stage IV (Mature)

This stage consists of large and turgid ovaries visible through the exoskeleton, from yellow to green intense. There are a few previtellogenic oocytes and mature oocytes (Mo, 150–200 μm), which are characterized by conspicuous cortical rods (CR) located at the cell periphery (Supplementary Fig. S3, Fig. 6–4A/4B).

#### Stage V (Degenerating or Spent)

Gonads are small, flaccid, and invisible through the exoskeleton. Ovaries have a resorbed appearance and included atretic or degenerating oocytes (Ao), with irregular shape and surrounded by granulosa cells (Gc; Supplementary Fig. S3, Fig. 6–5A/5B).

### Lipid Profile

**Figure 7:**
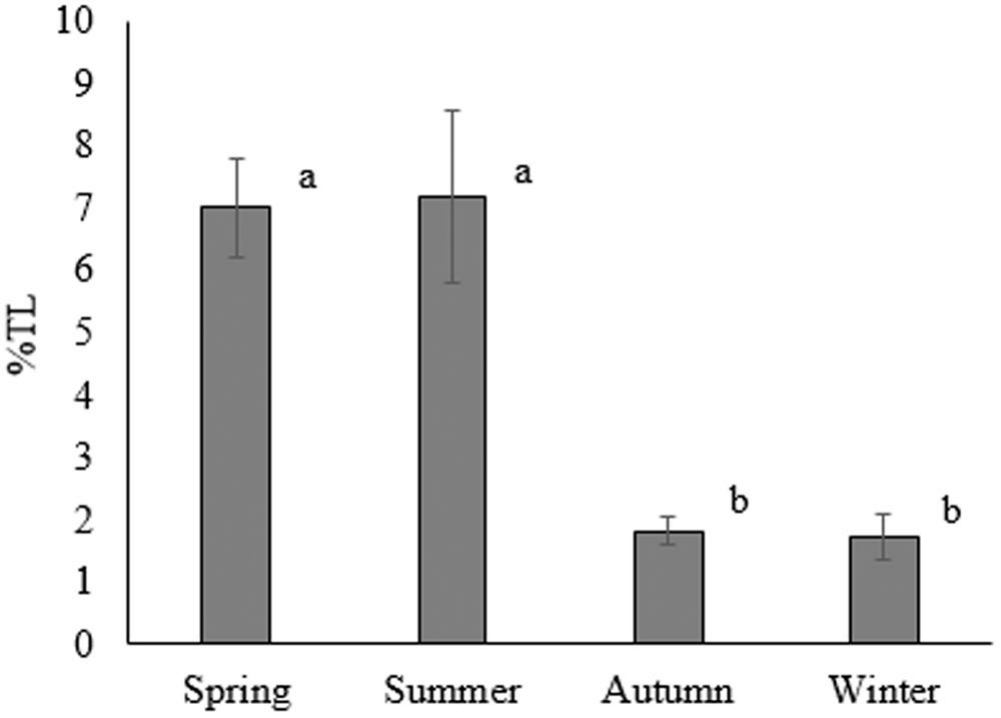
Total lipids concentration (%±SD, Standard Deviation) in P. kerathurus female gonads samples, in different seasons, from February 2015 to January 2016 (April 2015 excluded). Superscript letters indicate significant differences (p < 0.05).

From February 2015 to January 2016 (except for April when samples were not collected due to their seasonal migratory behavior) total lipids of gonad samples varied from minimum mean values of 1.8–1.7% dry weight (dw) in Autumn and Winter, to maximum mean values of 7.0% and 7.2% dw in Spring and Summer, respectively (Fig. 7). Whereas total lipid content remained more or less constant in the period February-September, a significant drastic decrease was recorded in the period October-January, with a reduction of about 75%.

Supplementary Table S1 shows the complete lipid profile of gonad samples of prawns collected in different months. Annual fluctuations were found for almost all FAs, with statistically significant differences between months. Minor FAs, with a percentage <1%, were excluded from the statistical analysis, because they were present in a not significant quantity. Concerning the most important SFAs, the highest values were recorded in Summer for myristic (14:0) and palmitic (16:0) acids, and in Autumn/Winter for margaric (17:0) and stearic (18:0) acids. Total SFAs ranged between 21.6% in May to 34.8% in July, with the highest values in Summer, and decreasing values from October until May. Between MUFAs, the most represented show the highest values in Summer (such as palmitoleic acid, 16:1n7 and oleic acid, 18:1n9), and in late Winter/Spring (*cis*-vaccenic acid, 18:1n7). Total MUFAs ranged between 22.2% in February to 31.8% in September, with the highest values in Summer. The essential fatty acid (EFA) 20:5n3 shows the highest values in Spring, and the lowest ones in Summer, whereas the other EFA 22:6n3 shows the highest values in June/July, and the lowest ones from November to December. Overall, total n-3 PUFAs ranged from 27% in September to 39.7% in May, with the highest values from February to June, and the lowest from July to September. n-6 PUFAs ranged from 5.8% in August to 16.6% in February, with the highest values in February/March and very low values from June to September.

Statistical analysis revealed significant correlations between GSI and some SFA, MUFAs, n-6 PUFAs and n3/n6 ratio (Supplementary Table S1). In more detail, myristic acid (14:0), palmitic acid (16:0), total SFAs, palmitoleic acid (16:1n7), oleic acid (18:1n9), total MUFAs, and n3/n6 ratio, showed a significant positive correlation with GSI, while margaric acid (17:0), heptadecanoic acid (17:1n7), eicosapentaenoic acid (20:5n3), linoleic acid (18:2n6), arachidonic acid (20:4n6), n6 PUFAs and total PUFAs showed a significant negative correlation with GSI (Supplementary Table SI).

To better understand the relationships between GSI, percentage of Total Lipids and FAs composition, a multivariate analysis (Principal Component Analysis, PCA) was performed to reduce the dimensionality of the data set in few components, that summarize the information contained in the overall data-set. GSI and the percentage of total lipids (*vs* dw, %TL) in gonads were included in the data-set, together with FAs with a percentage >1%.

Applying Principal Component Analysis to the data set (11 observations, 23 variables), it is possible to extract three significant, cross-validated principal components (Table 1). Together, they account for 90.8% of the variability in the original data. Table 1 shows the variance explained and the component loading matrix for the first three principal components extracted (PC1, PC2 and PC3). The higher the absolute value of a standardised coefficient, the more significant was the related selected variable. The first component is associated to GSI (positive coefficient), positively correlated with 14:0, 16:0, 16:1n7, 18:1n9, and total SFAs and MUFAs, as well as n3/n6 ratio, and negatively correlated with n6 PUFA (such as 20:4n6 and 18:2n6), and the n3 PUFA 20:5n3, as well as total PUFAs (negative coefficients). The second component is dominated by the percentage of Total Lipids, positively correlated with the 22:6n3, 18:1n7 and n3/n6 ratio (negative coefficients), and negatively correlated with saturated fatty acids such as 15:0, 17:0, 18:0, and the monounsaturated 17:1n7. GSI dominated the third component, that highlights the relationships between this index and some FAs, such as 15:0, 16:1n7, 20:2n6 (negative coefficients), and 18:0, 20:1n9, 22:6n3 (positive coefficients). This component explains only 7.21% of the variance, so we not considered this component in the discussion.

**Table 1:** Principal Component Analysis. Eigenvalues, explained and cumulative variance, loadings of the variables for the first three PCs.

	Principal Components		
	1	2	3
***Variance explained***			
Eigenvalues	12.27	6.940	1.658
% of variance	53.34	30.18	7.21
Cumulative %	53.34	83.54	90.75
***Factor loadings***			
GSI	**0.231**	−0.176	**0.248**
%TL	0.046	−**0.336**	−0.171
14:0	**0.266**	−0.038	0.030
15:0	0.120	**0.277**	−**0.279**
16:0	**0.273**	0.014	−0.054
16:1n7	**0.255**	−0.079	−**0.276**
17:0	−0.205	**0.258**	−0.030
17:1n7	−0.073	**0.338**	−0.239
18:0	−0.030	**0.305**	**0.361**
18:1n9	0.222	0.184	0.102
18:1n7	−0.126	−**0.292**	−0.194
18:2n6	−**0.243**	−0.093	−0.222
20:1n9	−0.213	−0.104	**0.271**
20:2n6	−0.046	−0.239	−**0.483**
20:4n6	−**0.241**	0.184	−0.004
20:5n3	−**0.273**	−0.045	−0.041
22:6n3	−0.033	−**0.321**	**0.265**
SFAs	**0.269**	0.104	0.048
MUFAs	**0.270**	0.038	−0.191
PUFAs	−**0.277**	−0.077	0.055
n3/n6	0.183	−**0.254**	0.199
n3	−0.215	−0.236	0.062
n6	−**0.253**	0.159	−0.039

The Analysis of Similarity (ANOSIM) for test the differences between the four seasonal FAs data highlighted statistical significant differences (p = 0.0074) with separation between level (R = 0.5689). The pairwise test indicated high separation level between summer *vs* winter and summer *vs* autumn (for all of them R = 1).

The Similarity Percentage Analysis (SIMPER) for all the seasonal pooled data highlighted a set of nine FAs that explain more than 90% of the cumulative dissimilarity between factors; in particular three PUFAs (20:4n6, 18.36%; 20:5n3, 17.22%; 22:6n3, 10.67%) explain 46.25% of the cumulative dissimilarity, followed by three SFAs (16:00, 14:00, 18:00) explaining 24.57% and three MUFAs (16:1n7, 18:1n9, 18:1n7) explaining the remaining 19.81% of the dissimilarity between factors. The seasonal pairwise Similarity of Percentage Analysis showed a contribution of two PUFAs as the major suppliers of dissimilarity in all comparison, ranged from 29.83% between winter *vs* autumn (20:5n3, 20:4n6) and 42.72% in spring *vs* autumn (22:6n3, 20:4n6; Supplementary Table S2).

## Discussion

Within the present study valid information on reproductive biology of *P. kerathurus* were obtained. In more detail, a sex-ratio biased towards female was observed and a 30.7 mm of CL size at first sexual maturity was estimated for females. This estimated CL size was lower respect to what reported in the literature: 40.7 mm in Thermaikos Gulf (N. Aegean Sea) by Kevrekidis & Thessalou-Legaki^47^, and 45.5 mm of CL for the same species in the South-Eastern coast of Italy by Lumare *et al*.^48^.

Presence of spermatophores adhering to *telycum* in females have been strongly connected with seasonality and size of the specimen, indicating summer season as the best period for insemination, but, unlike what was reported in Thermaikos Gulf by Kevrekidis & Thessalou-Legaki^47^, a percentage >50% of inseminated females was only recorded in summer months. Not statistically significant inter-annual variation was observed during the sampling season for the monthly sex ratio and monthly frequency of spermatophore.

Ovarian development was accompanied by changes in color (due to differences in carotenoid content) and size^20–22^, mainly related to the storage of macromolecules needed for the future offspring.

The ovarian maturation of crustaceans is characterized by an important accumulation of carotenoids, suggesting a role in their reproduction. Carotenoids play an important role not only during embryogenesis, as suggested by Goodwin^23^, Dall *et al*.^24^ and Liñán-Cabello *et al*.^25^, but also as a source of provitamin A^49–51^ and as antioxidants^52,53^. This work highlighted the most relevant macroscopic changes during gonadal maturation. The immature and early maturing ovaries changed from a milk/ivory, tiny and translucent appearance, during pre-spawning months (GSI at about 3–8%), to a yellow/greenish, thick aspect, clearly visible through the exoskeleton, in late maturing and mature stages, before the spawning phase (GSI at about 10–14%). After the most conspicuous spawning peak (in this work July), the ovaries returned back to the similar early maturing stage appearance, with whitish/ivory color, partially emptied (GSI at about 6%), reaching up to a completely empty ovaries, thin and translucent, in the spent-recovering stages, few months after spawning (GSI at about 1%; Supplementary Fig. S3). In crustaceans, oogenesis is characterized by the deposition of yolk in the oocyte, essentially the high-density lipoglycoprotein vitellin, frequently associated with carotenoids^54,55^. In fact, the vitellin of crustaceans is a lipo-glyco-carotenoprotein. Successful of gonadal maturation is genereally dependent on the diet^56–58^, especially for providing essential lipids necessary for the correct ovarian development for various shrimp species^37–40,42–44^. It is well-established that inadequate maternal nutrition affects maternal fecundity and egg viability in marine and freshwater fish and crustacean^59–61^. Lipid accumulation is strictly related to oocyte maturation which is usually characterized by morphological changes detectable by histological analysis. A typical five stages ovarian development was observed in this study. Oocytes increased their diameter from 34 µm in previtellogenic stage, up to 200 µm in mature stage, when they were characterized by conspicuous cortical rods in the cell periphery.

The size increase of the oocytes was correlated with an increase in total lipids as already reported for many penaeid species, including *P. kerathurus* ^38,62^. Total lipids increased from 1.7% in winter to 7.0% in spring and to 7.2% in summer. Consequently, the GSI ranged from 0.8 (December) to 11.2 (July), confirming July as the reproductive peak for this species (summer, from May to September).

**Figure 8:**
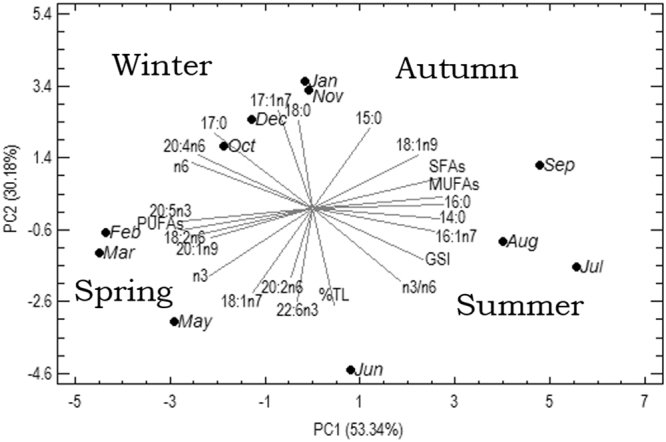
Biplot of Principal Component Analysis (PCA): relationships between GSI, percentage of Total Lipids and FAs composition (FAs < 1% were excluded; 11 observations, 23 variables).

From the biplot reported in Fig. 8, showing *loadings* and *scores* plots simultaneously, a relevant effect of seasonality was highlighted for this species. In particular, PC1 and PC2 showed that seasonality explained about 84% of the variability of all data-set: an annual cycle from February 2015 to January 2016 can be noted. The percentage of total lipids was high from February to March, and remained substantially constant, apart from a peak in June, but FAs composition changed in relation to the different months considered, because of the ovarian development. In particular, in the period February-May, lipids were characterized by a high PUFAs content, particularly FAs of the n-3 series. The Analysis of Similarity highlighted statistical significant differences with separation between level, indicating differences between the seasonal FAs composition, particularly evident between summer *vs* winter and summer *vs* autumn.

The statistical analysis of Similarity Percentage revealed in general a pool of FAs that have been defined the highest dissimilarity between factors. In more detail, in each seasonal pairwise comparison, two PUFAs were identified as the most influential source of dissimilarity among the seasons.

For *P. japonicus*, Teshima and Kanazawa^63^ highlighted a high lipid content in the ovaries, these molecules decreased to low level at nauplius and zoea stages, then remained roughly constant during the subsequent stages to post-larval. The same study verified that phospholipids (PL) and triglycerids (TG) were the major lipid classes in the ovaries and larvae, suggesting an important role of that these lipid classes as energy source and as constituent of the cell membranes in the ovaries and during embryogenesis. In the present study, a clear differentiation in a pre-spawning and a post-spawning phase was detected. During the summer season, the mature oocytes accumulated FAs that were used during embryogenesis and early larval stages, while in the cold season total lipids decreased drastically and the gonad reached a quiescent state.

In this species approximately 65% of fatty acids of the total ovarian lipids were conveyed to eggs during spawning; the fatty acids stored as triacylglycerol (TAG) in the midgut gland could play an important reserve role in the case of prolonged starvation before or during maturation or moulting^64^. Middleditch *et al*.^65^ stated that the increase in PUFAs concentration in the TAG fraction of both ovary and midgut gland could reinforce the theory that long-chain fatty acids are necessary for vitellogenesis of penaeids.

In the present study, several aspects were taken into account, highlighting changing during the reproductive season of *P. kerathurus*. The number of inseminated females increased in spring-summer months, as well as gonad size and GSI, together with color change due to carotenoid accumulation. The oocytes during gametogenesis increased their diameter and their total lipid content, changing their composition. Based on the GSI and FAs trends it is reasonable to assume this species, as a double batch spawner. The first batch is more evident in July followed by a second one, less noticeable batch in August-September. The fractional spawning strategy could allow more smaller and immature eggs to mature at different times. Probably the high food availability allows a transfer of high energy reserves to eggs as an investment in progeny, consequently splitting the spawning to two asynchronous batches. This strategy should amplify the reproductive outcome, not only in terms of overcoming starvation conditions during larval stages, but promoting a better chance of overcoming unfavorable environmental conditions, such as high temperature, low food availability, hypoxia crisis or storm surges. The post-spawning phase was characterized by a reduction of previously indicated events, with gonads in a quiescent phase.

This species was characterized by a pronounced seasonal migration along the year. The specimens move from the shallow waters in summer season during the early life stage, to deeper waters during the cold season, and vice-versa starting from next spring. As suggested by several authors^66,67^ the bottom water of the Adriatic Sea basin is characterized by more stable and limited fluctuation of environmental parameters, such as temperature, salinity and dissolved oxygen, contrary to what observed in surface water (Supplementary Table S3). This would prove the theory about the seasonal migration of the species, essentially related to the dependency between osmoregulation capacity and water temperature. The lowering of water temperature would result in a reduction of osmoregulation, with the species moving from a high temperature and salinity fluctuating area to a more stable environment, the deep waters.

The present work introduces new aspects related to the species, such as a pronounced seasonality of FAs composition strongly related to their gonadal maturation. The reproductive event needs an extraordinary amount of energy, prerequisite as an investment in the progeny. Few studies about the diet composition of *P. kerathurus* ^68,69^ have demonstrated a shifting in preys during the seasons and during the life cycle, probably linked to their availability (Supplementary Table S3). Also the length of the day, a key factor that strongly affects the reproduction of the species^70^, showed a very pronounced fluctuation among the seasons, ranging between eight to fifteen hours per day^71^ at the latitude of the study area (Supplementary Table S3), though, the role played by light in natural environmental for this species in the study area is not so clear, if considering that the Italian side waters are characterized by high turbidity level. The results of present study pointed out physiological mechanisms exerted by *P. kerathurus* in order to face the reproduction, assuming that seasonal migration associated with different prey availability would be able also to support and promote a remarkable energy investment in the reproductive events.

## Conclusion

A multidisciplinary approach was used in the present study to have a better knowledge about *P. kerathurus* reproductive cycle. For the first time, a chemometric approach was applied to GSI data, total lipids and fatty acid composition of gonads of *P*. *kerathurus*, highlighting a relevant effect of seasonality for this species. These results, together with classical microscopical analysis and fisheries data, allowed to obtain a better characterization of the annual reproductive cycle of *P*. *kerathurus*.

These results could represent a step toward a better comprehension of the reproductive biological traits of the species, allowing decisions managers to formulate proper fisheries management objectives based on adequate knowledge, needed for a sustainable exploitation of the fishery resources.

## Methods

### Sampling

Wild caramote prawns were monthly sampled in the period August 2013–January 2016 to study the maturity and gametogenic cycle. A total of 39 samplings (22 scientific- and 21 commercial-fishing surveys) were carried out in the Northern and central Adriatic Sea, Italy (Supplementary Fig. S1). The selected species typically migrates from coastal towards offshore waters in late summer and come back to reproductive coastal waters in spring. Thus, *P. kerathurs* specimens were collect from bottom trawlers in the period autumn-winter and by small scale artisanal fisheries in spring.

### Prawn Measurement

The ratio of population was estimated as the number of males divided by the total number of males and females collected. The observed sex ratio was tested for deviation from an expected 1:1 sex ratio using a binomial test^72^.

The carapace length (CL) of each specimen was measured from the eye orbit to the rear dorsal end of the carapace using a Vernier calliper with an accuracy of 0.01 mm (Supplementary Fig. S2). Body wet weight (ww) was measured to the nearest 0.1 g (electronic balance Mettler PL 3000). A Mann-Whitney test and a two-sample Kolmogorov-Smirnov test were than applied to the size frequency distributions of the different groups, i.e inseminated and not inseminated females, to assess statistical differences. The Analysis of Variance (one-way ANOVA) was applied to monthly sex ratio and monthly frequency of spermatophore in order to verify possible inter-annual variation of data during the sampling period.

Females were dissected and ovaries were gently removed and weighted at 0.0001 g (electronic analytical balance Mettler Toledo ML 204).

The GSI was expressed as the percentage of the ovarian weight in relation to the total body weight^29^ and calculated as:

1 $$\mathrm{GSI}=\frac{Weight\,gonad}{Weigh{t}_{total}-Weigh{t}_{gonad}}\times 100$$

Size at first maturity (CL50) was assessed based on the presence of spermatophores in female. CL50 was estimated by logistic equation as described by King^73^.

### Histology

After dissection, ovaries were preserved in Dietrich solution (distilled water, 95% ethanol, 40% formaldehyde and acetic acid) for histological analyses. Histological analyses were carried out on 31 females to evaluate gonadal development throughout the reproductive season and to validate the macroscopic stage attribution.

All samples, conserved in the fixative for at least 20 days, were embedded in paraplast, cut in transverse serial sections (7 µm) and mounted on slides. An increasing alcohol concentration protocol was adopted for dehydration. Slides were then stained with Harrys’ hematoxylin and eosin^74^. Histological sections were examined with a Leica DM 4000 microscope at 5–100× and 5–200× magnification with image analysis software LAS – Leica Application Suite.

### Lipid Profile

#### Sample Treatment

From February 2015 to January 2016, a total of fifty-five specimens from commercial fisheries of *P*. *kerathurus* were selected, five specimens per month, and their ovary intended to define lipid composition. Ovaries were homogenized and lyophilized (lyophilized by sublimation to −80 °C in high vacuum) until constant weight. Lipids were extracted through microwave extraction (MARS-X, 1500 W, CEM, Mathews, NC, USA) with petroleum ether and acetone in a ratio of 2:1 (v/v, Carlo Erba, Milano, Italy)^75^. Methyl esters of fatty acids (FAMEs) were obtained from total lipid extraction adding 1% of 2 M sodium methylate in methanol (obtained by methanol, Baker, Philipsburg, NJ, USA, and sodium methoxide for synthesis ≥97%, Merck, Hohenbrunn, Germany). The methyl ester of nonadecanoic acid (19:0, 99.6%, Dr. Ehrenstorfer GmbH, Germany) was used as internal standard IS^76^.

#### FAMEs Analysis

Methyl ester of fatty acids analysis were performed with gas chromatograph Agilent-6890 equipped with an Agilent-5973N quadrupole mass selective detector. A CPS ANALITICA CC-wax-MS (30 m × 0.25 mm ID, 0.25 μm film thickness) capillary column was used to separate FAMEs. The instrumental parameters were optimized using a 37-Component FAME mix (≥99%, Supelco, Bellefonte, PA, USA). Experimental condition of the GC/MS analyses were as in Canonico *et al*.^77^. Main ions fragment were recorded and identification of fatty acids was achieved using the NIST reference mass spectra database (NIST, Mass Spectral Database 02, National Institute of Standards and Technology, Gaithersburg, MD 2002) MS search 2.0a (NIST, 2002, NIST, Ringoes, USA). The analyses were performed in triplicate.

The estimated limits of detection (LOD) and limits of quantification (LOQ), calculated as in Truzzi *et al*.^76,78,79^, ranged for each FAME from ~4 μg mL^−1^ to ~22 μg mL^−1^, and from ~13 μg mL^−1^ to ~66 μg mL^−1^.

#### Statistical analysis

To compare FAs composition between months, the one-way ANOVA test, followed by the Multiple Range Test (Wayne, 2005) was performed after testing the homogeneity of variance with Levene’s test. Significant differences were evaluated at the 95% confidence level. Principal Component Analysis (PCA) was carried out on standardized data; significant components were obtained through the Wold cross validation procedure^80^. ANOVA test and PCA were performed using Statgraphics Plus 5.1. The non-parametric multivariate analysis were performed in order to test for differences between seasonal grouped FAs data. The Analysis of Similarity (ANOSIM; Bray-Curtis Similarity Index^81^, 9999 permutation) and Similarity Percentage Analysis (SIMPER; Bray-Curtis distance/similarity measure) were applied according to Clarke^82^.

## Electronic supplementary material

Supplementary Information
