# Supplementary material for: A multidisciplinary approach to study the reproductive biology of wild prawns
Source: Sci Rep. 2017 Dec 1;7:16781. doi: 10.1038/s41598-017-16894-1 (PMC5711900; doi:10.1038/s41598-017-16894-1)
Supplement: Supplementary file 1 — Supplementary Information [file 41598_2017_16894_MOESM1_ESM.pdf]

A MULTIDISCIPLINARY APPROACH TO STUDY THE REPRODUCTIVE BIOLOGY OF WILD PRAWNS.

Bolognini, L.*[1], Donato, F.[1], Lucchetti, A.[1], Olivotto, I.[2], Truzzi, C.[2], Randazzo B.[2], Antonucci, M.[2], Illuminati, S.[2], Grati, F.[1]

[1] National Research Council – Institute of Marine Science, Largo Fiera della Pesca, 60125 Ancona, Italy

[2] Department of Life and Environmental Sciences, Università Politecnica delle Marche, Via Brecce Bianche, 60131 Ancona, Italy

* Corresponding author: Luca Bolognini, E-mail address: luca.bolognini@an.ismar.cnr.it

Phone: +39 071 20 78 846, Fax: +39 071 55 313

Supplementary Table S1. Annual variations in FAs composition (% vs total FAs) in the ovary of *P. Kerathurus* females at different stages of ovarian development. Each value is the mean ±SD of five separate prawn samples analysed in triplicate for each. Within each row, superscript letters indicate significant differences (p<0.05). On the right column the correlation index r with GSI (*p< 0.5; **p<0.01). In grey lipids below 1% of the total FAs.

Lipids	Feb	March	May	June	July	Aug	Sept	Oct	Nov	Dec	Jan	ANOVA, p value	GSI correlation r	
12:0	0.03±0.01	0.31±0.1	0.04±0.01	0.61±0.2	0.59±0.5	1.17±0.5	0.73±0.3	0.15±0.1	0.16±0.1	0.09±0.03	0.16±0.1			
14:0	0.66±0.2^{a}	1.61±0.5^{b}	0.65±0.2^{a}	2.83±0.9^{d}	4.54±0.2^{e}	5.90±0.3^{f}	4.27±0.1^{e}	1.57±0.1^{b}	2.03±0.1^{b,c}	2.32±0.2^{c,d}	2.09±0.2^{b,c}	0.0000	0.72167	*
15:0	0.89±0.3	1.06±0.3	0.93±0.1	0.96±0.3	1.09±0.1	1.30±0.7	1.56±0.3	1.48±0.3	1.62±0.1	1.19±0.9	1.54±0.1	0.2496	-0.04993	
16:0	11.8±0.8^{a,b}	11.7±0.6^{a,b}	10.9±0.7^{a}	15.7±0.3^{e}	19.5±0.7^{f}	15.6±0.7^{e}	18.6±0.6^{f}	12.8±0.6^{b,c}	15.0±1^{d,e}	13.8±0.8^{c,d}	15.0±0.9^{d,e}	0.0000	0.81022	**
17:0	2.36±0.6^{c,d}	1.78±0.2^{b,c}	2.31±0.4^{c,d}	1.14±0.2^{a,b}	1.06±0.5^{a}	1.34±0.7^{a,b}	1.52±0.1^{a,b}	2.27±0.5^{c,d}	2.60±0.1^{d}	2.42±0.1^{c,d}	2.37±0.1^{c,d}	0.0001	-0.84941	**
18:0	7.00±1.3^{c,d}	4.82±0.6^{a}	6.39±0.6^{b,c}	5.60±0.4^{a,b}	6.07±0.7^{a,b,c}	7.00±1.0^{c,d}	5.46±1.1^{a,b}	6.45±0.5^{b,c}	8.28±0.8^{d,e}	8.34±0.3^{d,e}	8.51±0.9^{e}	0.0001	-0.38955	
20:0	0.13±0.1	0.10±0.1	0.11±0.1	0.31±0.2	0.31±0.1	0.38±0.1	0.29±0.1	0.18±0.1	0.19±0.2	0.21±0.1	0.21±0.2			
21:0	0.04±0.01	0.04±0.01	0.02±0.01	0.05±0.01	0.06±0.01	0.07±0.01	0.06±0.01	0.02±0.01	0.04±0.01	0.03±0.01	0.04±0.01			
22:0	0.11±0.2	0.04±0.01	0.09±0.01	0.10±0.5	0.14±0.1	0.13±0.2	0.11±0.1	0.04±0.01	0.06±0.01	0.06±0.01	0.07±0.02			
Total SFAs	23.2±0.3^{b}	21.6±0.6^{a}	21.9±0.8^{a,b}	27.4±0.8^{d}	33.4±0.9^{f}	33.0±0.7^{f}	32.7±0.6^{f}	25.0±0.6^{c}	30.0±1^{e}	28.4±0.9^{d}	30.0±1^{e}	0.0000	0.63789	*
14:1n5	0.02±0.01	0.03±0.01	0.02±0.01	0.11±0.2	0.26±0.2	0.43±0.2	0.30±0.1	0.03±0.01	0.04±0.01	0.02±0.01	0.07±0.01			
16:1n7	5.12±0.7^{a}	7.66±0.4^{d}	5.11±0.9^{a}	7.95±0.5^{d}	11.4±0.4^{f}	9.45±0.4^{e}	11.6±0.8^{f}	6.54±0.3^{b,c}	7.38±0.4^{c,d}	5.17±0.4^{a}	6.24±0.8^{b}	0.0000	0.81738	**
17:1n7	1.75±0.1^{b,c}	1.75±0.4^{b,c}	1.80±0.3^{b,c}	1.08±0.1^{a}	1.21±0.5^{a,b}	1.36±0.7^{a,b}	2.19±0.1^{c,d}	2.50±0.6^{d}	2.56±0.1^{d}	2.33±0.2^{c,d}	2.68±0.2^{d}	0.0001	-0.65259	*
18:1n9	8.73±0.9^{a}	9.22±0.8^{a,b}	8.63±0.1^{a}	10.3±0.7^{c}	12.1±0.4^{d}	12.8±0.6^{d}	12.9±0.9^{d}	10.8±0.5^{c}	9.94±0.3^{b,c}	10.3±0.6^{c}	10.9±0.3^{c}	0.0000	0.60859	*
18:1n7	4.54±0.7^{b,c}	5.04±1.1^{c}	4.69±0.7^{b,c}	4.25±0.3^{a,b,c}	4.03±0.8^{a,b,c}	3.71±0.2^{a,b}	3.35±0.4^{a}	3.27±0.2^{a}	3.80±0.8^{a,b}	3.46±0.1^{a}	3.31±0.6^{a}	0.0164	-0.05463	
20:1n9	1.90±0.4	1.54±0.4	1.98±0.3	1.67±0.6	1.39±0.3	1.38±0.4	1.29±0.4	1.66±0.4	1.24±0.4	1.80±0.3	1.51±0.1	0.3139	-0.49494	
22:1n9	0.07±0.01	0.04±0.01	0.06±0.02	0.11±0.1	0.12±0.1	0.10±0.1	0.12±0.2	0.18±0.1	0.04±0.01	0.19±0.1	0.04±0.01			
Total MUFAs	22.2±0.3^{a}	25.3±0.4^{c}	22.3±0.4^{a}	25.5±0.7^{c}	30.5±0.6^{d,e}	29.4±0.8^{d}	31.8±0.6^{e}	25.0±0.4^{b,c}	25.0±0.5^{b,c}	23.4±0.5^{a,b}	24.7±0.4^{b,c}	0.0000	0.7458	**
18:3n3	0.16±0.3	0.26±0.1	0.17±0.2	0.56±0.1	0.28±0.2	0.35±0.2	0.28±0.1	0.23±0.2	0.20±0.1	0.15±0.1	0.18±0.2			
20:3n3	0.17±0.2	0.16±0.1	0.16±0.1	0.34±0.1	0.21±0.1	0.22±0.1	0.15±0.1	0.14±0.1	0.13±0.1	0.13±0.1	0.13±0.1			
20:5n3	23.6±0.4^{e}	23.7±0.5^{e}	23.3±0.4^{e}	17.7±0.7^{b}	12.9±0.6^{a}	17.1±0.5^{b}	12.4±0.5^{a}	20.5±0.7^{d}	19.0±0.4^{c}	19.8±0.7^{c,d}	19.1±1^{c}	0.0000	-0.79611	**
22:6n3	13.9±0.3^{b}	15.6±0.7^{c}	15.9±0.4^{c}	21.6±0.7^{d}	16.3±0.3^{c}	14.0±0.3^{b}	14.2±0.7^{b}	14.4±0.3^{b}	11.8±0.8^{a}	12.6±0.5^{a}	12.0±0.3^{a}	0.0000	0.55351	
n-3 PUFAs	37.9±0.5^{f}	39.7±1.3^{g}	39.5±0.5^{g}	40.1±0.7^{g}	29.6±0.7^{b}	31.7±0.6^{c,d}	27.0±0.5^{a}	35.3±0.7^{e}	31.1±0.9^{c}	32.6±0.8^{d}	31.3±1.1^{c,d}	0.0000	-0.32474	
18:2n6	1.29±0.4	1.37±0.1	1.21±0.6	1.20±0.2	0.69±0.3	0.81±0.6	0.83±0.1	1.08±0.5	1.18±0.4	0.98±0.5	1.05±0.2	0.5598	-0.60527	*
18:3n6	0.03±0.01	0.08±0.01	0.03±0.01	0.13±0.1	0.09±0.01	0.10±0.03	0.08±0.01	0.08±0.01	0.07±0.01	0.05±0.01	0.08±0.01			
20:2n6	1.16±0.4^{a}	2.36±0.5^{b}	1.18±0.4^{a}	1.43±0.3^{a}	1.22±0.2^{a}	1.17±0.5^{a}	1.22±0.3^{a}	1.07±0.2^{a}	0.97±0.3^{a}	1.20±0.1^{a}	1.02±0.2^{a}	0.0037	0.09655	
20:3n6	0.24±0.2	0.30±0.1	0.25±0.4	0.33±0.1	0.22±0.1	0.22±0.1	0.19±0.2	0.27±0.3	0.30±0.2	0.22±0.1	0.22±0.3			
20:4n6	13.9±0.6^{f}	9.18±0.6^{c}	13.4±0.8^{f}	3.82±0.4^{a}	4.01±0.1^{a}	3.51±0.2^{a}	5.96±0.2^{b}	12.1±0.7^{e}	11.2±0.4^{d}	13.0±0.4^{f}	11.4±0.8^{d,e}	0.0000	-0.87721	**
n-6 PUFAs	16.6±0.2^{g}	13.3±0.6^{d}	16.1±0.4^{f,g}	6.90±0.5^{b}	6.24±0.1^{a,b}	5.81±0.3^{a}	8.28±0.2^{c}	14.6±0.5^{e}	13.7±0.4^{d}	15.5±0.4^{f}	13.8±0.8^{d}	0.0000	-0.8997	**
16:2n7	0.12±0.1	0.15±0.1	0.14±0.1	0.13±0.1	0.14±0.1	0.14±0.1	0.14±0.1	0.11±0.1	0.13±0.1	0.13±0.2	0.11±0.1			
Total PUFAs	54.7±1.6^{d}	53.1±1^{d}	55.7±1.6^{d}	47.2±1.7^{b,c}	36.0±2.4^{a}	37.6±2.7^{a}	35.5±1.5^{a}	49.9±1.3^{c}	44.9±1^{b}	48.2±0.9^{c}	45.2±1.4^{b}	0.0000	-0.72189	*
n3/n6	2.28±0.1^{a}	2.98±0.3^{b,c}	2.46±0.1^{a,b}	5.81±0.1^{e}	4.75±0.4^{d}	5.44±0.6^{e}	3.27±0.1^{c}	2.42±0.06^{a,b}	2.28±0.7^{a}	2.11±0.2^{a}	2.27±0.3^{a}	0.0000	0.82984	**

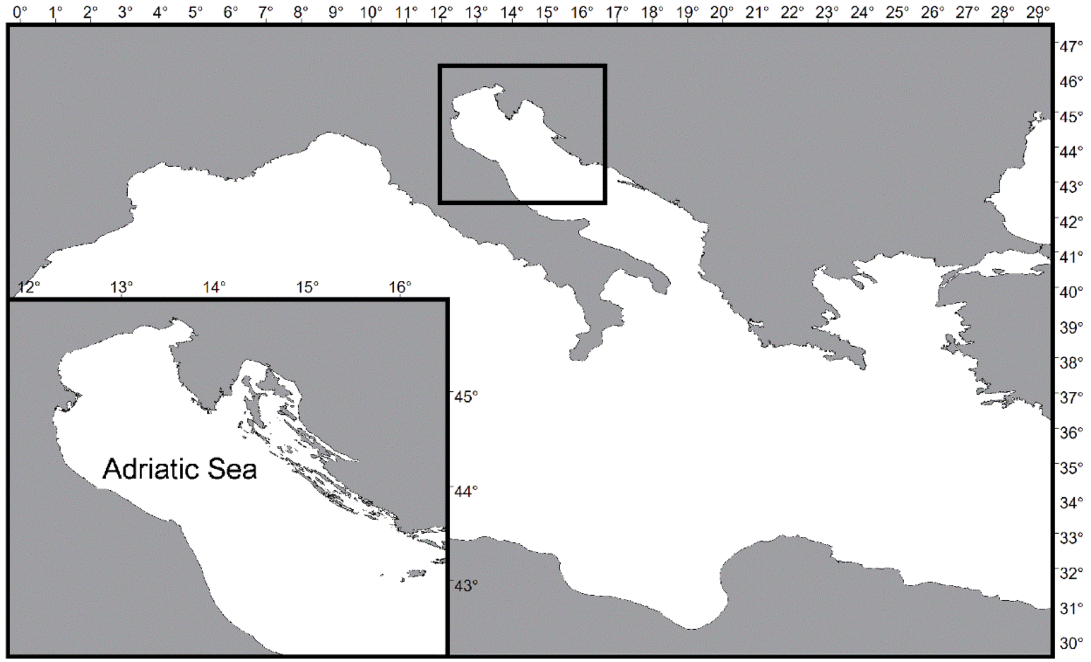

Supplementary Fig. S1. P. kerathurus sampling area in northern and central Adriatic Sea, Italy (map created by QGIS ver. 2.18).

Quantum GIS Development Team (2016). Quantum GIS Geographic Information System. Open Source Geospatial Foundation Project. http://qgis.osgeo.org

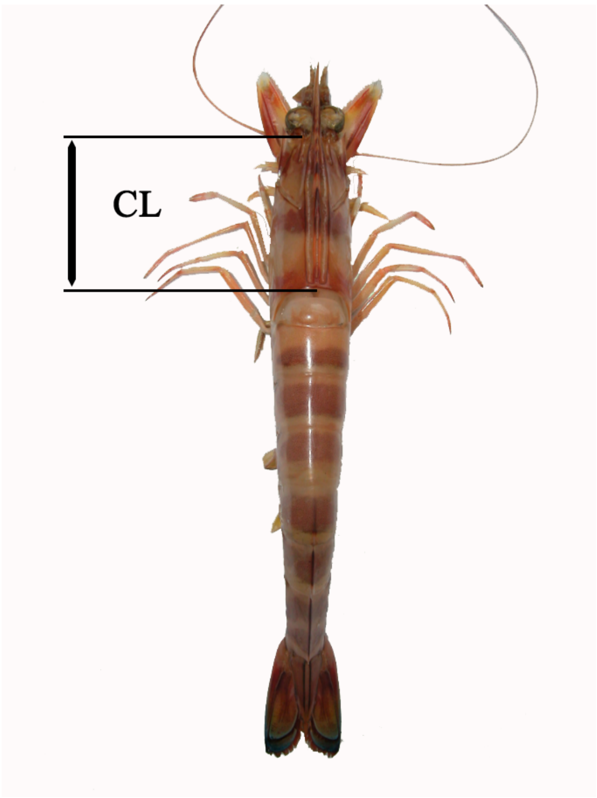

Supplementary Fig. S2. Schematic representation of P. kerathurus measurement recorded: carapace length (CL; Picture: Lucchetti A.).

Supplementary Fig. S3. Macroscopic temporal variation of female gonad of P. kerathurus, from May to November (CL= carapace length, mm; TL= total length, cm; GSI= gonado-somatic index).

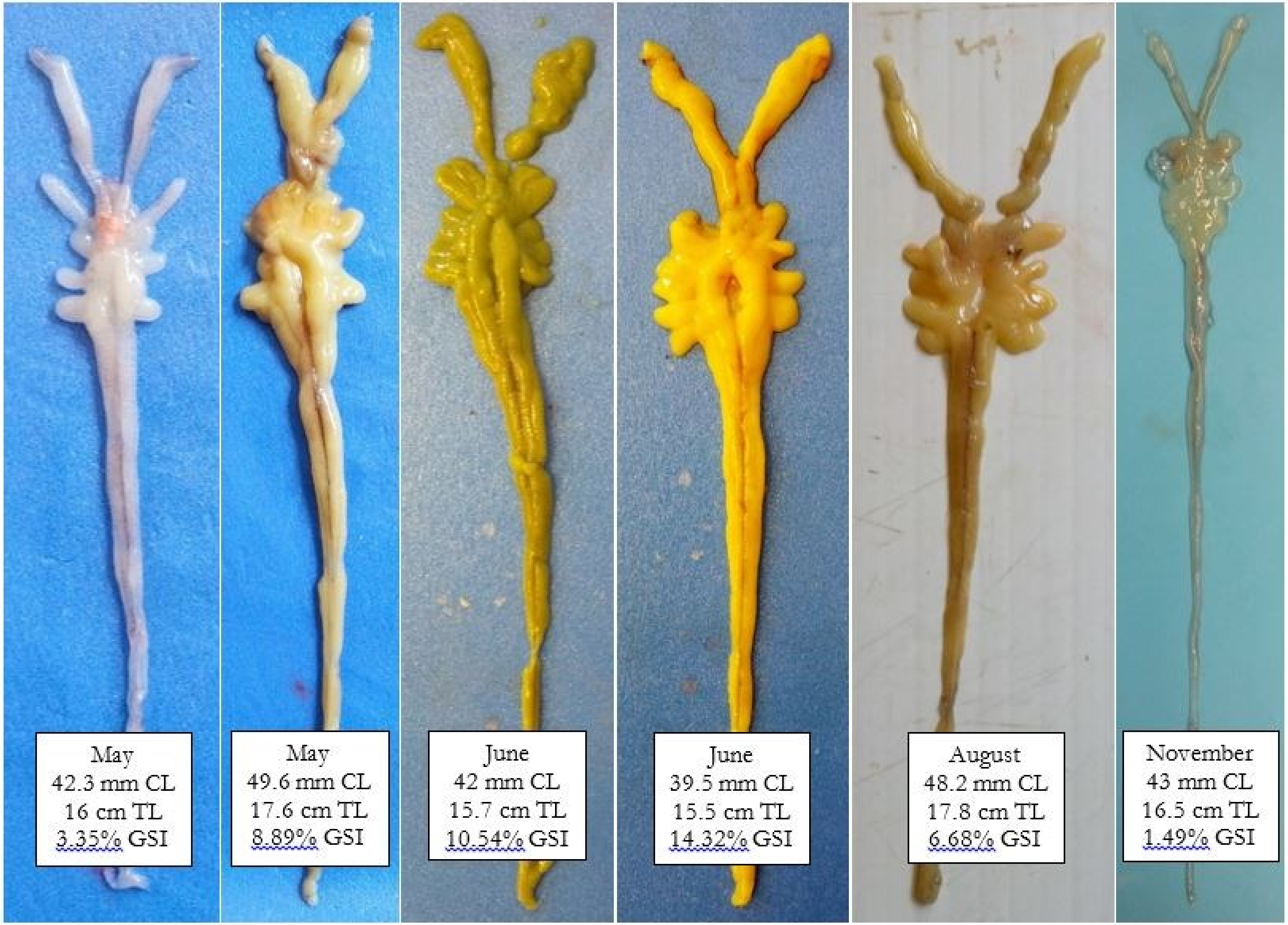

(Picture: Bolognini L.)

Supplementary Table S2. Seasonal pairwise comparison of percentage contribution of fatty acids explaining dissimilarity between factors.

Fas	Winter Vs Spring	Winter Vs Summer	Winter Vs Autumn	Spring Vs Summer	Spring Vs Autumn	Summer Vs Autumn	All pooled data
14:00	4.64	9.07	4.18	9.18	4.37	8.65	7.43
15:00	1.08	0.80	2.27	1.08	1.95	0.70	1.149
16:00	10.22	13.31	11.39	13.4	9.64	11.9	12.02
17:00	2.65	2.26	1.92	1.77	2.89	3.31	2.514
18:00	6.65	3.952	10.08	1.87	6.81	4.82	5.122
16:1n7	6.04	11.76	7.166	12.44	5.71	13.15	10.37
17:1n7	2.78	1.757	3.48	1.424	4.11	2.59	2.476
18:1n7	2.76	2.25	5.91	2.24	3.85	0.97	2.592
18:1n9	4.40	7.84	6.99	9.10	4.01	6.65	6.85
20:1n9	1.07	0.77	1.69	1.36	1.21	0.85	1.074
20:5n3	12.92	21.01	18.04	18.46	11.25	16.62	17.22
22:6n3	20.95	4.37	10.68	11.8	23.42	6	10.67
18:2n6	0.57	1.20	1.224	1.24	0.50	0.89	0.99
20:2n6	2.10	1.21	3.14	0.37	0.93	0.38	1.15
20:4n6	21.12	18.4	11.79	14.19	19.3	22.47	18.36

Supplementary Table S3. From the top to the bottom: water temperature (° C), salinity (PSU), dissolved oxygen (ml L^{-1}), caramote prawn pray composition in the Adriatic Sea and length of the day (hours) in the middle portion of the basin.

Parameter	Author	Level	WINTER	SPRING	SUMMER	AUTUMN
Temperature (°C)	*Lipizer et al. 2014*	Surface	< 9; >13	>18; < 16	23	<16; >18
		<-50 m	<13.5		<14.0; >15.0	>16
		<-100 m		>13.5	>13.5	>13.5
		<-200 m	<12; >13.5			
	Zavatarelli et al. 1998	Surface	>8.37; <13.73	>14.91; <16.03	>18.50; <20.51	>14.12; <17.11
		Deep	>9.24; <13.21	>11.45; <13.17	>11.45; <13.17	>11.40; <13.17
		MLIW	>12.69; <13.71	>13.41; <13.77	>13.75; <14.29	>13.75; <15.36
Salinity	*Lipizer et al. 2014*	Surface	<37; >39	<37	<37	
		<-50 m	<38.30; >38.6			
		<-100 m		>38.6	>38.6	>38.6
		<-200 m	<38.5; >38.6			
	Zavatarelli et al. 1998	Surface	>37.01; < 38.54	>35.87; < 38.51	>35.82; <38.46	>37.08; <38.44
		Deep	>37.95; <38.63	>38.49; <38.63	>38.45; <38.63	>38.44; <38.65
		MLIW	>38.49; <38.63	>38.51; <38.66	>38.55; <38.67	>38.56; <38.70
Dissolved Oxigen (ml L^{-1})	*Lipizer et al. 2014*	Surface		>6	<5.5	>6.0
		<-50 m	<5.4	<5.6	<5.5	<5.4
		<-100 m				
		<-200 m	>5.2			
	Zavatarelli et al. 1998	Surface	>5.52; <6.56	>5.48; <5.87	>4.94; <5.38	>5.32; <5.46
		Deep	>5.08; <6.09	>5.15; <5.24	>5.01; <5.10	>4.99; <5.01
		MLIW	>5.14; <5.49	>5.11; <5.51	>4.98; <5.44	>4.95; <5.28
Prey	*Prato et al. 2010*	Juvenile			Anphipods> Bivalvs> Gastropods= Crustaceans	
	Karani et al. 2005					Crustaceans
	Prato et al. 2010	Adult	Anellids> Molluscs> Foraminifers	Anellids= Molluscs= Foraminifers	Molluscs> Anphipods> Annelids	Anphipods> Molluscs> Annelids
	Karani et al. 2005					Molluscs> Crustaceans
Length of the Day 43°37'N 13°31'E (hours)	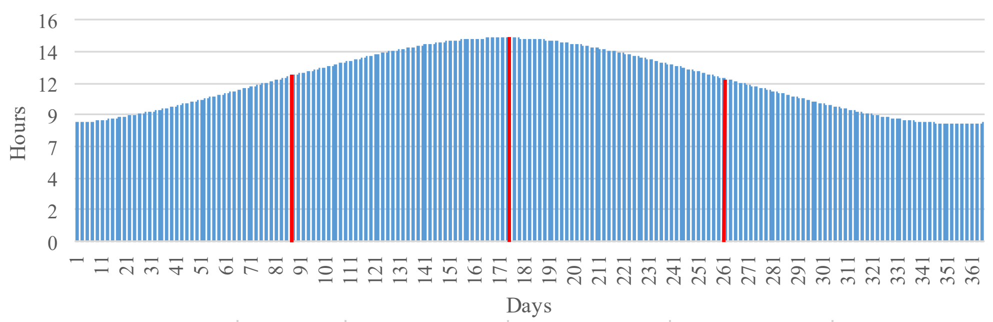					

Based on:

Karani, I. et al., 2005. Diet Composition of the Penaeid Shrimp, Melicertus Kerathurus (Forskål, 1775) (Decapoda, Penaeidae) in the Aegean Sea. Crustaceana, 78(4), pp.385–396.

Lipizer, M. et al., 2014. Qualified temperature, salinity and dissolved oxygen climatologies in a changing Adriatic Sea. Ocean Science, 10(5), pp.771–797.

Prato, E. et al., 2011. Alimentazione e predazione in natura di Melicertus kerathurus. In La risorsa Crostacei nel Mediterraneo: ricerca, produzione e mercato. Consorzio Nazionale di Ricerca per la Gambericoltura, Legnaro, pp. 86–96.

www.timeanddate.com. Available at: https://www.timeanddate.com/sun/italy/ancona?month=12&year=2016 [Accessed October 16, 2017].

Zavatarelli, M. et al., 1998. Climatological biogeochemical characteristics of the Adriatic Sea. Journal of Marine Systems, 18, pp.227–263.
